# Peste des petits ruminants in Africa: Meta-analysis of the virus isolation in molecular epidemiology studies

**DOI:** 10.4102/ojvr.v86i1.1677

**Published:** 2019-03-26

**Authors:** Samuel E. Mantip, David Shamaki, Souabou Farougou

**Affiliations:** 1 Department of Animal Health and Production, University of Abomey-Calavi, Abomey Calavi, Benin; 2 Viral Research Division, National Veterinary Research Institute, Vom, Nigeria

**Keywords:** peste des petits ruminants virus, lineages, isolates, molecular, epidemiology, vaccine, sheep, goat

## Abstract

Peste des petits ruminant (PPR) is a highly contagious, infectious viral disease of small ruminant species which is caused by the peste des petits ruminants virus (PPRV), the prototype member of the *Morbillivirus* genus in the Paramyxoviridae family. Peste des petits ruminant was first described in West Africa, where it has probably been endemic in sheep and goats since the emergence of the rinderpest pandemic and was always misdiagnosed with rinderpest in sheep and goats. Since its discovery PPR has had a major impact on sheep and goat breeders in Africa and has therefore been a key focus of research at the veterinary research institutes and university faculties of veterinary medicine in Africa. Several key discoveries were made at these institutions, including the isolation and propagation of African PPR virus isolates, notable amongst which was the Nigerian PPRV 75/1 that was used in the scientific study to understand the taxonomy, molecular dynamics, lineage differentiation of PPRV and the development of vaccine seeds for immunisation against PPR. African sheep and goat breeds including camels and wild ruminants are frequently infected, manifesting clinical signs of the disease, whereas cattle and pigs are asymptomatic but can seroconvert for PPR. The immunisation of susceptible sheep and goats remains the most effective and practical control measure against PPR. To carry out PPR vaccination in tropical African countries with a very high temperature, a thermostable vaccine using the rinderpest lyophilisation method to the attenuated Nigeria 75/1 PPR vaccine strain has been developed, which will greatly facilitate the delivery of vaccination in the control, prevention and global eradication of PPR. Apart from vaccination, other important questions that will contribute towards the control and prevention of PPR need to be answered, for example, to identify the period when a susceptible naïve animal becomes infectious when in contact with an infected animal and when an infectious animal becomes contagious.

## Introduction

Peste des petits ruminant (PPR) is a highly contagious, infectious viral disease of small ruminants species which is caused by the peste des petits ruminants virus (PPRV), the prototype member of the *Morbillivirus* genus in the family Paramyxoviridae and the order Mononegavirales (Gibbs et al. 1979). Currently there are seven known members of the genus *Morbillivirus*, which include the measles virus (MV), rinderpest virus (RPV), PPRV, canine distemper virus (CDV), phocine distemper virus (PDV), cetacean morbillivirus (CeMV) and feline morbillivirus (FMV) (Woo et al. 2012). CeMV is further grouped into three genetically distinct viruses: the porpoise morbillivirus (PMV), pilot whale morbillivirus (PWMV) and dolphin morbillivirus (DMV). Peste des petits ruminants virus has a linear negative-stranded RNA genome that consists of 15 948 nucleotides and the PPRV genome that carries six transcriptional units or genes; each encodes for a contiguous and non-overlapping protein except the P gene, which also expresses C and V non-structural proteins (Mahapatra et al. 2003). The six PPRV genes encode six structural proteins, namely, the nucleocapsid protein (N), phosphoprotein (P), fusion protein (F), matrix protein (M), hemagglutinin-neuraminidase protein (HN), large protein (L) and two non-structural proteins, namely C and V, in the order of 3’-N-P/C/V-M-F-HN-L-5’ (Bailey et al. 2005; Mahapatra et al. 2006; (Rahaman et al. 2003). The molecular epidemiology of the PPR virus, based on the sequence comparison of a small region of the F gene (322 nt [nucleotideprotein or basepair]; Forsyth & Barrett 1995), the N gene (255 nt; Couacy-Hymann et al. 2002) or the H gene (298 nt; Kumar et al. 2014), has defined the existence of four distinct lineages, that is, lineages I–IV of the virus (Banyard et al. 2010; Dhar et al. 2002; Kwiatek et al. 2011; Libeau, Diallo & Parida 2014; Shaila et al. 1996). The nomenclature of lineages I and II is slightly different for analyses depending on whether the N or F gene is being considered (lineage II of the F gene data is considered to be lineage I based on N gene data and vice versa). However, recently a comparison has been made between genetic data derived from the F, N and H genes and the partial N gene sequence revealed a greater variability than the F and H genes; therefore, lineage classification according to the N gene is considered the most accurate way to type novel isolates, as this region is more divergent between lineages and between isolates of the same lineages (Kumar et al. 2014). All PPRV strains belong to a single serotype, but the different strains have been grouped into four distinct lineages. Historically, lineages I–III have been found in Africa and numbered according to the apparent spread of the virus from West Africa (lineages I and II) to East Africa (lineage III). Lineage IV was mainly restricted to the Middle East and Asia, with a few exceptions of lineage III being reported in Yemen and Oman, and mixed lineages of lineages III and IV in the United Arab Emirates and Qatar. However, lineage IV has now established its presence all across the PPR-endemic areas, with frequent outbreaks in Africa, by replacing other lineages (Kwiatek et al. 2011; Libeau et al. 2014; Mantip et al. 2016; Muniraju et al. 2014; Woma et al. 2015) (Mantip et al. 2016). Recently, the evolution of PPRV and its relationship with its prototype RPV has been suggested using molecular phylogenetic techniques with virus full genome sequence data. Bayesian phylogenetic studies found that rRPV is more closely related to measles virus than to PPRV (Furuse, Suzuki & Oshitani 2010; Pomeroy, Bjornstad & Holmes 2008). The origin of ancestral PPRV and its relationship with other morbilliviruses have recently been estimated using Bayesian analysis of the complete virus genome sequences (Kumar et al. 2014). A Bayesian phylogenetic analysis of all four viral lineages mapped the time to the most recent common ancestor at the beginning of the 20th century, a few decades before the first recorded description of the virus in 1942. Lineage III PPRV is proposed to have been the first to have diverged (Parida et al. 2015). The estimated probability for the root location of an ancestral virus and individual lineages was determined as being Nigeria for PPRV, Senegal for lineage I, Nigeria/Ghana for lineage II, Sudan for lineage III and India for lineage IV (Muniraju et al. 2014).

The life cycle of PPRV is 6 hours – 8 hours in cultured cells (Kumar et al. 2014). The initial step of infection of PPRV starts with the binding of the virus to the host cell and delivery of the nucleocapsid into the host cell cytoplasm; this plays an important role in the pathogenesis of the virus and susceptibility to the host (Kumar et al. 2014). The first interaction of the host and pathogen (attachment) is mediated by the binding of the virus to the cell receptors through its H protein (Kumar et al. 2014). Similar to MV and CDV, PPRV has two natural cellular receptors: the signaling lymphocyte activation molecule (SLAM) or CD150 protein and nectin-4. The signaling lymphocyte activation molecule is exclusively expressed on immune cells (lymphocyte, macrophages and dendritic cell surface but not on epithelial cells), while nectin-4 is the epithelial cell receptor, but is not expressed in lymphocytes and dendritic cells (Adombi et al. 2011; Munir, Munir & Zohari 2013; Pawar et al. 2008). The existence of these two different receptors may explain why morbilliviruses are both lymphotropic and epitheliotropic (Kumar et al. 2014). Following the release of nucleocapsid from the viral envelope, viral transcription starts in the cytoplasm. The RdRp present in the infecting virion initiates synthesis of the mRNA as well as complementary RNA (cRNA). The RdRp of all paramyxoviruses is believed to attach at the genomic promoter (GP) on genomic RNA, from where the transcription begins (Baron et al. 2017; Barrett, Banyard & Diallo 2005). The process of assembly and release of the morbillivirus including PPRV is not well understood. Like other enveloped viruses, paramyxoviruses also form virus particles when all the structural components of the virus, including viral glycoproteins and viral RNPs, have assembled at selected sites on the membranes where virions bud, then pinch off to achieve particle release (Kumar et al. 2014), allowing the transmission of infections to new cells and hosts (Harrison, Sakaguchi & Schmitt 2010).

Apart from the endemic presence of PPR in sub-Saharan Africa from the past decades, in recent years, field data and laboratory findings have confirmed the dramatic spread of PPR towards the south of Africa, affecting Gabon, the Democratic Republic of Congo, Somalia, Kenya and Tanzania (Swai et al. 2009). An outbreak of PPR was reported for the first time in October 2012 in Angola and in July 2015 in Zambia (Baron et al. 2016). The risk of PPR introduction is now high for neighbouring countries with major sheep/goat populations, such as the Republic of South Africa and Mozambique. In addition to the spread in Africa, many Asian countries are now infected, including China. After an initial identification in Tibet in 2007 (Wang et al. 2009), this country experienced a major PPR epizootic in 2013–2014 (Wang et al. 2009).

Peste des petits ruminant affects sheep and goats, although goats are often more severely affected than sheep (Lefevre & Diallo 1990). However, variable seroprevalence has been observed in sheep and goats after an outbreak (Abraham et al. 2005; Ayari-Fakhfakh et al. 2011; Ozkul et al. 2002; Swai et al. 2009). Various factors may explain these differences: livestock management practices, host density, strain virulence (Couacy-Hymann et al. 2007) as well as host species and breeds (Diop, Sarr & Libeau 2005). For instance, Sahelian goats are considered more resistant than Guinean dwarf goats, while Alpine goats are very sensitive after experimental infections (Hammouchi et al. 2012). Peste des petits ruminant is not considered as pathogenic in cattle, domestic and wild African buffaloes (*Syncerus caffer)* although 10% or more of these species may seroconvert when exposed to PPRV in enzootic regions (Abraham et al. 2005; Couacy-Hymann et al. 2007; Ozkul et al. 2002). In a nationwide serological survey recently implemented in Senegal (2015), seroprevalence rates as high as 80% were observed in regions where both cattle and small ruminants were abundant, without any reported clinical sign in cattle (Baron et al. 2016). Conversely, high case fatality rates (96%) were reported in India in domestic buffaloes (*Bubalus bubalis*) and the disease was experimentally reproduced in these animals (Govindarajan et al. 1997). In addition, PPR has been suggested to occur as a disease in camelids; a respiratory syndrome was the main sign in Ethiopia and Sudan (Khalafalla et al. 2010; Roger et al. 2000). Other wild ruminants, including representatives of the Gazellinae, Tragelaphinae Caprinae subfamilies, may show serious illness and mortality when infected with PPRV from neighbouring small ruminant populations. In specific conditions, wildlife may play an important role in PPR epidemiology, as was seen in the Arabian Peninsula (Kinne et al. 2010). The incubation period of the disease is typically 4–6 days, although it may range between 3 and 14 days. During the acute stage of the disease, animals show pyrexia (up to 41 °C) that may last for 3–5 days and that can be accompanied by depression, anorexia and dryness of the muzzle. Watery nasal and lachrymal discharges gradually become mucopurulent with excessive salivation. Erosive lesions formed in the oral cavity may become necrotic. In severe cases of the disease, these necrotic lesions progress with the appearance of a deposit of fibrin (caseous deposit) on the tongue. In later stages of the disease, animals develop diarrhoea and coughing with laboured, abdominal breathing. Finally, the animal may become dyspnoeic, suffering from progressive weight loss and emaciation, ultimately leading to its death. In some cases, particularly in mild infections, animals may convalesce, returning to a pre-infection health status within 10–15 days of infection. The morbidity rate can reach 100% with a high case fatality rate in the acute form of the disease (Pope et al. 2013).

At an early stage of infection, virus excretion is very high in the exhaled air. By analogy with RPV, this probably allows non-contact transmission over at least a few metres (Idnani 1944). Nasal and ocular discharges, saliva and faeces also contain large amounts of viral antigen (Abubakar et al. 2012). In goats, PPRV–RNA or antigen is excreted in the faeces for at least 2 months after a natural infection (Abubakar et al. 2012; Ezeibe et al. 2008), although it is not known if this is an infectious virus. Because PPRV is quickly inactivated in the environment, its transmission most often occurs by direct contact between infected and healthy animals. However, indirect transmission through recently (within hours) contaminated material cannot be excluded and should be considered in epidemiological models and risk-based control measures (Baron et al. 2016). Because of its rapid spread in immunologically naïve flocks, a common belief is that PPRV can only persist in large populations and only if new susceptible hosts (newborn, migrating or purchased animals) are available (Wamwayi et al. 1995).

The economic losses that are associated with PPR can be substantial. Peste des petits ruminant has severe implications on the livelihoods of pastoralists and farmers across the world. The world’s populations of 2.1 billion small ruminants are critical assets for poor rural households in developing countries, providing quality protein, milk, nutrition, fertiliser, wool and fibre, as well as income opportunities and financial flexibility. Around 80% of those animals are found in regions where PPR, is present (FAO 2013). The virus is now present or expected to exist in more than 70 countries and it keeps spreading, and appearing in areas or countries where it did not exist before, almost every year, and causes an estimated $1.5 to $2 billion financial loss each year. The control, prevention and eradication of PPR are linked to other global challenges such as poverty alleviation, food security and nutrition, and resilience building, in particular for smallholder farmers and women.

## History of peste des petits ruminants research in Africa

In Africa, PPR has often been confused with the closely related RPV, the history of which dates back to as far as the late 4th century AD (Curasson 1932; Henning 1956). Rinderpest virus is predominantly a disease of large ruminants and is linked historically with devastating epidemics, often with very high mortality rates. In contrast to the extensive historical data available on RPV, the first recorded description of PPRV was in 1942 when Gargadennec and Lalanne identified a disease closely related to RPV in the Ivory Coast in West Africa. Early observations suggested that the disease was not transmissible from small ruminants to in-contact cattle and this led them to assume it was a novel virus distinct from RPV (Gargadennec & Lalanne 1942). Because of the similarity between RPV and PPR, they have common names such as ovine rinderpest, goats’ plaque, pseudo-rinderpest, pneumoenteritis complex, stomatitis-pneumoenteritis and plague of small ruminants or KATA; however, because of the official discovery of PPR in the Ivory Coast which is a French-speaking colony, pest des petits ruminants (PPR), which means plague of small ruminants, is commonly used worldwide. In 1956, it was shown by Mornet and colleagues that PPRV and RPV are closely related antigenically (Pastoret, 2006). In 1979, PPRV was classified as a morbillivirus under the family Paramyxoviridae and the order Mononegavirales (Gibbs et al. 1979). Peste des petits ruminant was first isolated in sheep cell culture in 1962 (Gilbert, Y. & Monnier, J., 1962) and was observed for the first time under electron microscope in 1967 (Bourdin & Laurent-Vautier 1968). Peste des petits ruminant has been an important disease of sheep and goats in Africa ever since its discovery and has therefore been a major focus of research in virtually all the veterinary research institutes and university faculties of veterinary medicine in Africa. Several key discoveries with regard to the nature of PPRV and its epidemiology, through serological and genetic methods, were carried out in African institutions that contributed towards the understanding and control of PPR. In 1962, Gilbert and Monnier in their effort to develop a homologous PPR vaccine carried out the first adaptation of PPRV in tissue culture when a cytopathic effect (CPE) of the virus was observed in primary sheep liver cells, manifested by the appearance of syncytia (Gilbert & Monnier 1962). Years later, PPRV was adapted in Vero cells and a homologous live attenuated PPR vaccine was developed. This was the first PPR vaccine that was developed in Africa for worldwide PPR vaccination by continuous passage of Nigeria 75/1 strain in Vero cells and is now commonly used in African countries. Based on circumstantial evidence, such as strong seasonal incidence and the fact that sheep/goats could be protected by vaccination and by avoiding low lying pastures, it was stipulated that outbreaks of PPR are always attributed to changes in weather, that is, either during the onset of the rainy season or during the dusty harmattan season. Once animals are infected by PPR, sooner or later they begin to manifest the clinical signs and symptoms of the disease and can easily transmit the virus to susceptible naïve populations, thereby leading to the spread of the disease in a particular population. Consequently, as the transmission of PPRV from virus-excreting infected to naïve animals mainly occurs through close contact, the most important sanitary preventive measure consists of restricting the importation of susceptible animals from infected areas to disease-free areas. Outbreaks can be controlled by stamping out infected specimens followed by disinfection of premises and compensation of affected farmers. However, as most of the PPR-endemic regions are in Africa, such drastic measures are difficult to implement. Thus, vaccination is the main means available for the effective prevention and control of PPR in those countries (Baron et al. 2016). The main characteristic of the pathogenesis of PPRV infection, as for other morbilliviruses, is the profound but transient virus-induced immunosuppression, with the consequence of increased susceptibility to opportunistic infections that impact the final outcome of the infection (Baron et al. 2014; Pope et al. 2013). This immunosuppression is a consequence not only of direct effects of virus multiplication in lymphoid cells but also of the different strategies developed by morbilliviruses to overcome the host protective response, such as interference with both the innate and induced immune responses. However, this immunosuppressive effect is transient and recovery from the disease is usually accompanied by the establishment of a strong, specific and long-term protective immune response (Servet-Delprat et al. 2003). Attenuated morbillivirus vaccines seem to have less immunosuppression capacity compared to wild-type viruses, but still induce a strong protective immunity (Sen et al. 2010). With that characteristic, the live attenuated rinderpest vaccine (Plowright & Ferris 1962) was one of the key factors in the success of the global rinderpest eradication programme. After Plowright and Ferris’ success, attempts were also made to develop a live attenuated PPR vaccine which, however, was unsuccessful (Benazet 1973; Gilbert & Monnier 1962). Following those failures, and considering the close antigenic relationship between RPV and PPRV, the live attenuated rinderpest vaccine was tested in small ruminants for protection against PPR. Despite the lack of a detectable PPRV- neutralising antibody, these animals resisted the PPRV challenge and the RPV vaccine was successfully used to protect small ruminants against PPR (Rowland & Bourdin 1970; Taylor & Abegunde 1979; Taylor, Al Busaidy & Barrett 1990). Continued research on the development of a homologous PPR live attenuated vaccine was finally successful in 1989, with the attenuation of the PPRV strain Nigeria 75/1 by means of serial passage in Vero cells (Diallo et al. 1989b). Several field trials of this vaccine conducted between 1989 and 1996 demonstrated its high efficacy in protecting sheep and goats against PPR. Anti-PPRV antibodies generated by vaccinated animals last for at least 3 years, equal to the effective economic life of the animals. During the development of this vaccine, different wild-type PPRV strains were used as challenge viruses and all failed to induce disease in vaccinated animals, demonstrating the efficacy of the vaccine against virulent PPRV strains of any lineage. The availability of a homologous vaccine for PPR was fortunate as the use of rinderpest vaccine in any animal species was forbidden once a country was declared free of rinderpest. Following this first success, other PPRV strains were successfully attenuated by serial passage in cell culture (Sen et al. 2010). Amongst them, the PPRV/Nigeria 75/1 (lineage II) and the PPRV/Sungri/96 (lineage IV) are currently the most used vaccines and have been extensively tested and validated, including the determination of full genome sequences (Diallo 2003, 2004; Diallo et al. 2007; Riyesh et al. 2011; Singh & Bandyopadhyay 2015; Singh et al. 2009). Like all paramyxoviruses, PPRV is heat sensitive. Field distribution of the live attenuated PPRV vaccines therefore needs a cold chain from the manufacturer to the point of delivery to the animal. Unfortunately, most of the countries where PPR is endemic are in regions of hot climate and have poor infrastructure with inconsistent electricity supply. This issue has been addressed by attempting to improve the freeze-drying procedures for the attenuated PPRV vaccines using various stabilisers (Riyesh et al. 2011; Saravanan et al. 2010; Sarkar et al. 2003; Silva, Carrondo & Alves 2011; Silva et al. 2014; Worrall et al. 2000). With such improvements, it has been possible to keep the vaccine at 45 °C for at least 14 days with minimal loss of potency (Worrall et al. 2000). One significant problem with the live attenuated PPRV vaccines is that animals that have received this vaccine cannot be distinguished serologically from animals that have been infected and recovered. This makes epidemiological serosurveillance of the disease impossible in areas where a vaccination programme has been or is being implemented. A way to combine vaccination and disease serosurveillance activities for better management of the disease would be the use of vaccines, enabling differentiation of infected and vaccinated animals (DIVA). With the advent of recombinant DNA technology, different approaches are being followed to develop vaccines that enable this differentiation, to allow countries to implement both vaccination and disease surveillance programmes at the same time (Diallo et al. 2007).

The development of *in vitro* systems for isolation and large scale propagation of PPRV in the 1960s paved the way for serological, antigenic and genetic studies of the virus as well as the development of diagnostic assays, thereby improving the understanding of the virus.

## Global recognition of peste des petits ruminants

Currently, PPR is the fastest expanding and potentially the most economically important disease of sheep and goats in many regions of the developing countries where these domestic animals play an integral and important role in sustainable agriculture and development (Baron et al. 2016). Peste des petits ruminant has spread so alarmingly during the last two decades that it has become a matter of concern for the Food and Agriculture Organization of the United Nations (FAO) and the International Organization Epizootic (OIE), which have now initiated efforts for its control and eradication (OIE 2015).

Peste des petits ruminants was first identified in Nigeria (West Africa) in 1942. It is currently believed to be endemic across much of West Africa (Banyard et al. 2010). West Africa includes 16 countries distributed over an area of approximately 5 million square kilometres. Almost all of these countries have experienced significant outbreaks of PPRV. In recent years, material submitted to Regional Reference Laboratories (RRLs) has confirmed the presence of either antibodies to the virus or the detection of viral nucleic acid in samples from Burkina Faso (2008), Ghana (2010), Nigeria (2007, 2013, 2014) and Senegal (2010) (Banyard et al. 2010). Peste des petits ruminants virus strains from both lineages I and II are currently circulating across West Africa although undoubtedly many outbreaks are not characterised at the molecular level. Characterisation of PPRV in Nigeria from a recent outbreak indicated that PPRV lineage IV is also circulating alongside PPRV lineage II in Nigeria (Mantip et al. 2016). East Africa is generally used to specifically refer to the area now comprising the countries of Kenya, Tanzania and Uganda but often includes Somalia, Djibouti, Ethiopia and Eritrea. Peste des petits ruminants virus is endemic across the majority of these countries, with genetic typing of the virus in 1996 determining a virus circulating in Ethiopia as belonging to lineage III (Banyard et al. 2010). Previous isolation of viruses that group in lineage III includes two isolates from wildlife in Oman (1983) and the United Arab Emirates (1986), an isolate from Sudan (1972) and an unexpected virus isolate from sheep in southern India (1992) (Banyard et al. 2010). More recently, the confirmation of endemicity of PPRV across East Africa has been shown through the detection of antibodies to PPRV in Kenya (1999 and 2009) and Uganda (2005 and 2007). Molecular tools have characterised some viruses as belonging to lineage III, with isolates being characterised in Sudan (2000), Uganda (2007) and most recently in Tanzania (2008 and 2010) (Banyard et al. 2010). Somalia was also affected by PPRV in 2006, with the central regions being most seriously affected (Nyamweya et al. 2009). Peste des petits ruminants virus was also seen in Ethiopia in 2008 and 2009 (Nyamweya et al. 2009). Despite the absence of molecular typing for the recent Kenyan and Somalian outbreaks, it is likely that the virus circulating in these areas is lineage III. Central Africa includes a core region of the African continent, namely, Burundi, the Central African Republic (CAR), Angola, Cameroon, Chad, Gabon, the Democratic Republic of the Congo (DRC) and Rwanda. Phylogenetic analysis has shown that lineage IV viruses are circulating across Central Africa (Banyard et al. 2010). Geopolitically, northern Africa includes seven countries or territories: Algeria, Egypt, Libya, Morocco, Sudan, Tunisia and the Western Sahara. Algeria, Morocco, Tunisia, Mauritania and Libya together are sometimes referred to as the Maghreb (or Maghrib), while Egypt is a transcontinental country by virtue of the Sinai Peninsula, which is in Asia. Historically, it is hypothesised that PPRV spread into North and East Africa from West Africa, moving up through trade routes through Sudan and Egypt and into the Middle East. Historically, PPRV had been detected in 1987 and 1990 in Egypt (Ismail & House 1990). Molecular typing has characterised a recent discovery of PPRV in Egypt as lineage IV (Banyard et al. 2010). With the transcontinental status of Egypt it is suggested that the remainder of North Africa was totally free from PPRV until recently when an extensive outbreak occurred in Morocco. In 2008, local veterinary services reported 257 outbreaks across 36 of Morocco’s 61 provinces. Despite low mortality and morbidity rates, this outbreak was of great significance because of commercial trade between Morocco and both Algeria and Spain. Genetic characterisation of the Moroccan virus classified it as a lineage IV virus (FAO 2009; Khalafalla et al. 2010).

In the Arabian Peninsula and the Middle East, the presence of PPRV was analysed in Saudi Arabia and it was concluded that the disease was not circulating in the country (Al-Naeem, Elzein & Al-Afaleq 2000). However, by April 2002 an outbreak with a case mortality rate of 100% was reported in sheep and goats (Housawi et al. 2004) and since then further serological surveys and outbreaks have been reported (Al-Dubaib 2009; El-Rahim et al. 2005; Housawi et al. 2004). Seroprevalence of both sheep and goats has also been reported in North Jordan (Al-Majali, Hussain & Amarin 2008) and in Lebanon with a seroprevalence of up to 48.6% being reported (Attieh, 2007). In the remaining areas of the Arabian Peninsula, lineage IV virus has been detected in a game reserve in the United Arab Emirates (Kinne et al. 2010) as well as in Qatar (2010). Interestingly, in Qatar, both lineages III and IV are circulating, with both lineages being isolated from goats in 2010 (Banyard et al. 2010). The situation in Qatar is further complicated by the recent detection of PPRV in wild deer populations. However, the role of sylvatic PPRV and potential transmission to domestic species remains unknown (De Nardi et al. 2012). Within Yemen, the most southern region of the peninsula, lineage III virus continues to circulate, with no introduction of lineage IV having been reported in Yemen or Oman (Banyard et al. 2010).

Peste des petits ruminants virus has been reported in Pakistan since 1991, with initial epidemics in the Punjab region being characterised by using PCR in 1994 (Amjad et al. 1996). Since then both the spread of the virus and an increase in reporting meant that PPRV has been documented on several occasions in Pakistan. Outbreaks that affected Afghan sheep (Bulkhi) occurred in the Punjab (Durrani et al. 2010), Lahore (Rashid, Asim & Hussain 2008) and Islamabad (Zahur et al. 2009). Currently, only the lineage IV virus has been identified in Pakistan (Banyard et al. 2010). Peste des petits ruminants virus is endemic across much of India, and an improvement in veterinary services, reporting networks and diagnostic capabilities across India has led to an increase in awareness of the disease (Banyard et al. 2010). The virus was first reported in southern India in 1987 (Shaila et al. 1989), where it seemed to remain for several years before spreading across the entire country and surrounding regions. Molecular characterisation of virus isolates from India shows that virtually all isolates analysed belong to lineage IV. More recent epidemiological studies of PPRV in India have characterised a number of closely related lineage IV viruses being present (Dhar et al. 2002). The virus continues to be reported periodically across India, with recent reports documenting the presence of PPRV in Rajasthan (Kataria, Kataria & Gahlot 2007) in the north, the Kolkata region in the east (Saha, Lodh & Chakraborty 2005), Karnataka and Maharashtra in the south-west (Chavran, Digraskar & Bedarkar 2009; Santhosh et al. 2009) and across the southern peninsula (Raghavendra et al. 2008). These reports reflect the endemicity of the disease across the entire country.

The presence of PPRV is poorly characterised across great stretches of the Middle East, with outbreaks in Iraq most frequently being reported. Detection of PPRV in Iraq dates back to 2000 where a virus causing high morbidity and low mortality rates was characterised (Barhoom, Hassan & Mohammed 2000). However, retrospective sero-analysis has shown that the virus was previously circulating in 1994 (Shubber et al. 2004). Iran has also had PPRV circulating for many years, with initial detection back in 1995, leading to the extensive spread of the virus across the country at a huge economic cost (Bazarghani et al. 2006). More recently in 2009, the virus from Iran has been characterised at the genetic level, being grouped with lineage IV viruses (Banyard et al. 2010). The recent detection of PPRV in areas of the Near East has highlighted the potential for spread of PPRV into areas that have never previously been documented. Tajikistan borders Afghanistan where PPRV is believed to be present and was previously reported in association with RPV outbreaks (Kwiatek et al. 2007). Peste des petits ruminants virus is also believed to be present in Kazakhstan, although only very few seropositive animals have been identified (Lundervold et al. 2004). The recent detection of PPRV in China was officially reported in the Ngari region of western Tibet in July 2007 (Wang et al. 2009). Previously, PPRV infection had been recognised in several countries bordering the south-western region of China, including India, Nepal (2009), Bangladesh (2000 and 2009), Pakistan (2004 and 2009) and Afghanistan (Banyard et al. 2010). All the recent virus strains detected in south-west Asia and the Middle East belong to lineage IV and the Tibetan isolate is of the same lineage and is closely related to isolates from both India and Tajikistan. The terrain of western and south-western Ngari permits uncontrolled animal movement, and a small ruminant trade exists between Tibet and the bordering nations such as India and Nepal. These factors suggest that animals from a neighbouring country in south-west Asia are the most likely source of PPRV in Tibet (Wang et al. 2009). Serological detection of antibodies to PPRV has also been detected in samples from Vietnam (Maillard et al. 2008) and samples submitted to RRLs from Bhutan have recently been typed as lineage IV virus (Banyard et al. 2010). These findings suggest that the virus may be present across a greater area than what is currently thought. It is possible that PPRV has spread into many other bordering countries, but the unfamiliarity of local human populations with the disease means that it may remain either unnoticed or be misdiagnosed as a different disease with similar clinical manifestations (Banyard et al. 2010).

Recent reports of PPRV in areas close to European borders have increased its profile both scientifically and in the media. While this devastating disease of small ruminants has continued to plague agriculture across Africa and Asia for many years, the threat of its spread into developed countries has greatly renewed research interest in the virus. The detection of PPRV in Turkey in 1996 raised initial awareness of the virus and questioned the potential for PPRV to spread across the rest of Europe (Ozkul et al. 2002). Indeed, there have been numerous reports of PPRV in Turkey, having now also been reported in Western Turkey, Bursa province (Yesilbag et al 2005) and Mugla and Aydin provinces (Toplu 2004) in the Aegean district. Throughout 2005, 78 separate outbreaks of PPRV were recorded across Turkey, with quarantine and vaccination being used to prevent further spread of the disease (Tufan 2006). In 2007, the first outbreak of fatal PPRV was reported in Kirikkale province, Central Anatolia (Kul et al. 2007), suggesting the spread of the virus into the central belt of the country, and in 2009 infection of sheep in the Middle and Eastern Black Sea region of Turkey was reported (Albayrak & Alkan 2009). These reports suggest that PPRV is present across much of Turkey and diagnostic samples received by RRLs have confirmed Turkish PPRV isolates as belonging to lineage IV (Banyard et al. 2010). Consequently, the Moroccan outbreak and the potential existence of as-yet-unidentified foci of PPRV infection across other territories in northern Africa also increase the threat of movement of infected animals into southern Europe. Historical exchanges exist between Morocco and Spain where both ovine and caprine populations are important. An increase in human population and in turn the small ruminant population, across such areas undoubtedly increases the risk of further emergence of PPRV across North Africa. It is therefore essential that Europe maintains surveillance of the disease in order to successfully contain the disease should it be imported (Minet et al. 2009).

## Peste des petits ruminants in Africa

Generally in Africa, PPR occurs throughout the year, but it has been documented in various reports that the prevalence of the disease varies according to climatic conditions and seasons. Obi et al. (1983b) in a study carried out in western Nigeria showed that PPR may be encountered almost throughout the year, with peaks in the wet months of June–August. They also observed that at the University of Ibadan Teaching and Research Farm, 192 (74%) cases recorded in goats between 1973 and 1980 were in April–July, while out of the five natural outbreaks that were investigated, three occurred in the wet season (June–July) and the remaining two were recorded in November–December. Wosu et al. (1990) in a study in the Nsuka area reported that PPR incidence was higher during the dry harmattan season (December–January) than in the rainy season, with a peak in April. They attributed this to the dry, cold and dusty harmattan with the accompanying poor nutrition at that time of the year. Seasonal occurrence of the disease was also observed by Whitney, Scott and Hill (1967) and Nduka and Ihemelandu (1973). Other factors that have been reported to affect the prevalence of PPR in Africa include the acquisition of animals from local markets, mass transportation of animals during major festivals (Bonniewell 1980), local husbandry and customary practices of renting males and all year-round kidding and constant introduction of new stock, leading to an increased number of susceptible animals (Obi et al. 1983b). During the observation of the first cases of PPR in Nigeria, it was thought that the disease was a problem of dwarf goats in the wet and humid southern parts of Nigeria. However, serological surveys in southern Nigeria by Obi et al. (1983a) and in Northern Nigeria by Gibbs et al. (1979) showed widespread infection in the country. Bourdin (1983) reported that the highly acute form of the disease most frequently seen in goats was rare in Sahelian sheep. Sheep and goats of all ages and sexes can be affected during an outbreak (Obi et al. 1983a). Bourdin reported that in goat populations, the highest incidence occurs in young animals between 1 and 24 months, with losses of up to 50%.

Consequently, it had been thought that PPRV is restricted in its host range, being confined to its natural infections to sheep and goats, with the latter species being more often affected than the former (Losos 1986). The isolation of PPRV from dorcas gazelle, which died of a natural infection in the United Arab Emirates (Furley, Taylor & Obi 1987), has broadened our understanding of the host range of the virus. In cattle, PPR was not shown to be pathogenic although there was seroconversion (Gibbs et al. 1979). The investigations on a new pathology of camels in Ethiopia and Sudan (Roger et al. 1997) have implicated PPRV in a natural outbreak of an acute febrile infection characterised by respiratory signs. Results of experimental transmission indicate that cattle and pigs do not suffer from clinical disease (Nawathe & Taylor 1979). Shamaki (2002) reported the probable role of wildlife (warthog) and camel in the epidemiology of PPR in Nigeria. Elaborately, lineage differentiation of PPR is determined by the sequence comparison of a small region of the F gene (Forsyth & Barrett 1995) or N gene (Couacy-Hymann et al. 2002), depending on the reaction components used by the testing laboratory. Historically, African isolates of PPRV were numbered lineages I–III according to the proposed spread of the virus from West Africa to East Africa. Following this nomenclature, the N gene primer sets (Couacy-Hymann et al. 2002) typed West African viruses from Senegal, Guinea, Guinea-Bissau, Ivory Coast and Burkina Faso as belonging to lineage I. The isolates derived from Ghana, Mali and Nigeria then formed lineage II and those detected in Ethiopia and Sudan were from lineage III. Data derived from the F gene material reversed the classification of lineages I and II isolates, and historically this difference has been maintained (Shaila et al. 1996). The current molecular characterisation of PPRV virus isolates divides them into four genetically distinct lineages: lineage I being represented mainly by Western African isolates from the 1970s and recent isolates from Central Africa; lineage II by West African isolates from the Ivory Coast, Guinea and Burkina Faso; lineage III by isolates from Eastern Africa, Sudan, Yemen and Oman; and lineage IV includes all viruses isolated from recent outbreaks across the Arabian Peninsula, the Middle East, Southern Asia and recently across several African territories. Consequently, data generated by PCR and sequencing are routinely used to construct phylogenetic trees for PPRV and ascribe different isolates to the different lineages in order to understand the spread and epidemiological movement of the virus (Dhar et al. 2002; Ozkul et al. 2002; Shaila et al. 1996).

## Research on peste des petits ruminants in Africa

The continuous incursion of PPRV into previously unaffected regions of Africa, the Middle East and Asia stimulated research interest in PPRV in Africa, and right from the identification of PPRV in Nigeria, different techniques have been employed to contribute towards the control and prevention of PPRV. The techniques and approaches used to improve PPRV isolation and molecular research and diagnosis right from inception include virus isolation, detection of viral antigen and nucleic acid sequencing as outlined below.

### Virus isolation

During the inception of PPRV research, the commonly used cell culture systems employed in PPRV isolation were primary lamb kidney and ovine skin tissues (Bourdin, P. & Laurent-Vautier, 1968) (Gilbert, Y. & Monnier, J., 1962)(Taylor, W. P. & Abegunde, 1979). The use of Vero cells was later employed (Hamdy et al. 1976). The sensitivity of PPRV isolation was then increased by growing the virus in lamb’s or goat’s kidney cells (Ali & Taylor 1984). Peste des petits ruminants virus has also been adapted to grow in other continuous cell lines including Madin-Darby Bovine Kidney epithelial cell line and Baby Hamster Kidney-21 fibroblast cell line (MDBK and BHK-21) (Diallo et al. 1989b). However, Vero cells derived from African green monkey kidney were the most widely used cell line for PPRV before the discovery of the goat or dog SLAM. Following the discovery of a protein receptor on the cell surface of morbillivirus susceptible hosts, the SLAM, also known as CD150, is used preferentially by wild-type morbilliviruses to bind to the host (Banyard, Baron & Barrett 2005; Baron 2005; Cocks et al. 1995; Cosby et al. 2002; Kruse et al. 2001; McQuaid & Cosby 2002; Ono et al. 2001; Sidorenko & Clark 1993; Tatsuo et al. 2000; Tatsuo, Ono & Yanagi 2001; Yanagi, Takeda & Ohno 2006). However, although lymphoid tissues are major sites of morbillivirus replication, it is also observed that they can infect and replicate in epithelial cells of other organs, such as the alimentary track epithelial cells, and lung and kidney cells by utilising other types of cell receptors (Hashimoto et al. 2002; Leonard et al. 2008; Plowright 1964; Takeuchi et al. 2003; Wohlsein et al. 1993; Wohlsein et al. 1995). The infection efficacy for those cells is up to 100–1000 times less than that of the lymphoid cells (Hashimoto et al. 2002; Takeda et al. 2007), but because they are easy to maintain in culture *in vitro*, they have been favoured preferentially for morbillivirus isolation. In the case of PPRV, primary cultures of bovine kidney, goat kidney, sheep kidney and lung cells have all been utilised for isolation and maintenance (Abu Elzein et al. 1990; Ali & Taylor 1984; Lefevre & Diallo 1990; Taylor & Abegunde 1979; Taylor, Al Busaidy & Barrett 1990). However, not only is the availability of primary cells becoming more problematic, their quality is also not guaranteed and there is considerable variation from batch to batch. These drawbacks in the use of primary cell cultures have stimulated the use of cell lines for PPRV isolation, in particular, the African green monkey kidney (Vero) cell line (Lefevre & Diallo 1990). Unfortunately, as with other morbilliviruses, PPRV isolation using these cells is inefficient: the likelihood of isolating the virus is very low, and even if it is successful, it often requires multiple, sequential blind passages and many weeks in culture before the development of any CPE can be observed (Abu Elzein et al. 1990; Albayrak & Alkan 2009; Gulyaz & Ozkul 2005; Lefevre & Diallo 1990). Following the identification of the SLAM as the main receptor used by MV, CDV and RPV wild-type strains (Banyard et al. 2005; Hsu et al. 2001; Tatsuo et al. 2000, 2001), Vero or CHO cells expressing human, dog or bovine SLAM proteins have been used as a highly efficient means of isolation and propagation of these morbilliviruses (Tatsuo et al. 2001; Seki et al. 2003; Tatsuo et al. 2001). Based on the discovery of SLAM as a possible protein for the isolation and propagation of PPRV, a CV1 cell line stably expressing the goat SLAM protein was developed for the isolation of PPRV (Adombi et al. 2011). This cell line, designated as CHS-20, is used to isolate PPRV from clinical specimens of sick sheep and goats with suspected PPR specimens collected in 2008 and 2009 from different locations in Nigeria and Cote d’Ivoire, respectively (Adombi et al. 2011). Preliminary tests carried out demonstrated a high sensitivity for isolating PPRV from pathological samples, indicating the recent use of SLAM in PPRV isolation and propagation in place of the previous methods.

### Agar gel immunodiffusion test

Agar gel immunodiffusion (AGID) was widely used to identify PPRV and it can detect 42.6% of ante-mortem and necropsy specimens (Abraham & Berhan 2001; Obi 1984). Agar gel immunodiffusion can be used to test the presence of both antigen and antibodies and can give results within 2 h – 4 h when rinderpest (RP) hyperimmune serum is used, while it needs 4 h – 6 h with PPR hyperimmune serum (Obi 1984). One of the important advantages of this test is that it is highly specific (92%), though it cannot differentiate between PPR and RP.

### Hyperimmune serum

Standard PPR anti-serum was made by immunising sheep with 5 mL of PPRV with a titre of 10^4^ TCID_50_ (50% tissue culture infective dose) per millilitre administered at weekly intervals for 4 weeks. The animals were bled 5–7 days after the last injection. Standard RP hyperimmune anti-serum is also effective in detecting PPR antigen (Abraham et al. 2005).

### Counter immuno-electrophoresis

Counter immuno-electrophoresis (CIEP) used the same principle as the AGID except that the gel is electrically charged to improve the sensitivity of the test (Abraham et al. 2005).

### Enzyme-linked immunosorbent assay for antigen detection

A monoclonal antibody-based sandwich enzyme-linked immunosorbent assay (ELISA) was found to be highly effective in the detection of antigen in tissues and secretions of infected goats (Saiki et al. 1988). Another format of antigen ELISA which was more widely used is immunocapture ELISA (Abraham et al. 2005). It gives a reliable result within 2 h in pre-coated plates and samples maintained at room temperature for a period of 7 days with no more than 50% reduction in response (Libeau et al. 1994). The immunocapture ELISA allows for a rapid differential diagnosis of PPR and RP viruses, and this is of great importance as the two diseases have similar geographical distributions and may affect the same animal species. The main advantages of this assay include rapidity, specificity, robustness and simplicity. It is suitable for routine diagnosis of PPR and RP from field samples such as ocular and nasal swabs.

### Complementary DNA probes

For the differentiation between PPR and RP, the use of [P^32^]-labelled complementary DNA (cDNA) probes derived from the N-protein gene of the two viruses had been described (Diallo et al. 1989b). It could differentiate between the two viruses without the need of virus isolation. cDNA directed against the matrix protein, fusion protein and phosphoprotein gene was found to cross-hybridise to a much greater extent and was not suitable for use as discriminating probes (Diallo et al. 1989a). Unfortunately, this hybridisation cannot be used widely because it requires fresh specimens and in addition to the short half-life of [P^32^], there is a constraint with the handling of isotopes. Therefore, probes using non-radioactive labels such as biotin (Pandey, Baron & Barrett 1992) or dioxin (Libeau et al. 1995) were developed. The biotin-labelled cDNA was found to be as specific as the one using the radioactive label and more rapid in differentiation between PPR and RP (Pandey et al. 1992). However, it was reported elsewhere that the expected sensitivity responsiveness had never been obtained using non-radioactive labels (Diallo et al. 1995).

### Reverse transcription polymerase chain reaction

Conventional serological techniques and virus isolation are normally used to diagnose morbillivirus infection in samples submitted for laboratory diagnosis. However, such techniques are not suitable for use on decomposed tissue samples; the polymerase chain reaction (PCR) has been proved invaluable for analysis of such poorly preserved field samples. Saiki et al. (1988) first demonstrated the efficiency of amplifying *in vitro* a selected sequence flanked by two oligonucleotide primers of opposite orientation. The method consists of repetitive cycles of DNA denaturation, primer annealing and extension by a DNA polymerase effectively doubling the target with each cycle, leading, theoretically, to an exponential rise in DNA product. When the PCR starting material is DNA, high purification of the nucleic acid is not necessary; therefore, the procedure is greatly simplified. These qualities have made the PCR one of the essential techniques in molecular biology today and have made it the most widely used laboratory technique in disease diagnosis. Since the genome of all morbilliviruses consists of a single strand of RNA, it must be first copied into DNA, using reverse transcriptase, in a two-step reaction known as reverse transcription/polymerase chain reaction (RT-PCR). Reverse transcription/polymerase chain reaction has been shown to be useful for the rapid detection of morbillivirus-specific RNA in samples submitted for laboratory diagnosis (Shaila et al. 1996). It has proved especially useful in identifying the new morbilliviruses found in marine mammals (Barrett et al. 1993). Both genus-specific and universal morbillivirus primer sets have been produced, which can be used to distinguish all known morbilliviruses (Forsyth & Barrett 1995). Two sets of primers have been made based on sequences in the 3’ end of N genes (messenger sense), which are the least conserved regions between the two viruses. They enable specific amplification of 300 base pair (bp) fragments for RPV and PPRV (Couacy-Hymann et al. 2002). Reverse transcription/polymerase chain reaction, using phosphoprotein (P) universal primer and fusion (F) protein gene-specific primer sets to detect and differentiate between PPR and RP, was described by some researchers (Barrett et al. 1993; Couacy-Hymann et al. 2002; Forsyth & Barrett 1995).

Advancement in modern technology, awareness and availability of molecular tools in Africa led to an increased interest in PPRV following RPV eradication; outbreaks of PPRV have been genetically typed, enabling molecular epidemiological assessment of lineage distribution across endemic areas of Africa. This in turn has led to speculation surrounding the emergence of a dominant lineage of PPRV in new areas (Kwiatek et al. 2011). Certainly, the recognition of the importance of PPR as an obstacle to the development of sustainable agriculture across Africa has led to the laboratory confirmation of PPR disease, with subsequent reporting of cases to the scientific press (Banyard 2016). The advent of molecular technologies over the last 20 years has led to studies into the genetic composition of different isolates of PPRV, which has fostered a greater understanding of the molecular epidemiology of PPR in Africa. From a genetic point of view, four distinct lineages of PPRV have been described based on genetic data derived from the nucleoprotein gene (Couacy-Hymann et al. 2002). Furthermore, the rapid development of sequencing technologies has revolutionised sequence generation from samples with the result that full genome data can be derived from samples with relative ease, enabling phylogeneticists to assess isolates based on any part of the genome (Banyard 2016). Most recently, the first Bayesian full genome assessment of PPRV has concluded that from an evolutionary perspective, PPRV emerged at the beginning of the 20th century, a few decades before the first recorded description of PPRV in 1942 (Muniraju et al. 2014). A Bayesian phylogenetic analysis of all PPRV lineages mapped lineage III PPRV as the first to have diverged from an ancestral virus. The estimated probability for the root location of an ancestral PPRV and individual lineages was determined as being Nigeria for PPRV as a whole, Senegal for lineage I, Nigeria/Ghana for lineage II, Sudan for lineage III and India for lineage IV. In recent years, PPRV has extended its boundaries southwards in Africa, as far as southern Tanzania (2008) and Zambia (2015) and the Democratic Republic of Congo and Angola (2012) (Banyard et al. 2010; FAO 2013). Peste des petits ruminant outbreaks have also been reported across North Africa, including Tunisia (2006), Morocco (2008) and Algeria (2011).

The hypothesis that dominant lineages may enter areas and overwhelm existing lineages of the virus is of great interest. Certainly, the extensive detection of lineage IV virus in Africa in recent years has led some to hypothesise that lineage IV is a dominant lineage that is replacing other lineages across different areas (Banyard 2016). Historically, the detection of lineage IV PPRV in Central Africa was the first detection of this lineage on the African continent (Banyard 2016). While reports of PPRV infection are frequently reported in the scientific literature and to the OIE, genetic characterisation is required to understand the epidemiology of the virus (Banyard 2016; Banyard, Baron & Barrett 2005). The expansion of lineage IV virus across Africa has been a slow process when considering reports of genetic characterisation of this lineage in different areas. Whether local extinction of other lineages occurs in Africa is unclear as reports where genetic data exist for local outbreaks are few (Banyard 2016). The reported expansion of lineage IV virus across Central and East Africa and the explosion of lineage IV virus between 2008 and 2012 across North Africa have further fuelled ideas surrounding the enhanced pathogenicity of lineage IV strains of the virus (Kwiatek et al. 2011). A recent study in Nigeria has demonstrated an extensive distribution of lineage IV virus alongside a fairly limited distribution of the lineage II virus that has been documented historically (Mantip et al. 2016; Woma et al. 2015). Whether this increased representation of lineage IV virus in different regions indicates a dominance of viruses from this lineage is unclear. Certainly, as with other morbilliviruses (Baron 2005; Barrett, Banyard & Diallo 2005), the determinants of virulence for PPRV remain almost completely undefined and as such potential lineage-driven dominance remains speculative, especially as many regions are unable to genetically type circulating viruses. In addition, recent reports of lineages I, II and IV in Uganda (Luka et al. 2012) and Tanzania (Parida et al. 2015), lineages II and IV in Nigeria (Mantip et al. 2016; Woma et al. 2015) and lineage III in Sudan suggest that other lineages of the virus remain present in the region despite the emergence of lineage IV (Banyard et al. 2010). The apparent expansion of Asian lineage IV across Africa is supported by a constant increase in the incidence of disease, suggesting an increase in virulence (Libeau, Diallo & Parida 2014). Virus lineages circulating in Africa is shown in Table 1.

**TABLE 1:** Lineages of peste des petits ruminants virus circulating in different countries of Africa, based on partial N/F gene sequence analysis.

Country	Year of first report	Lineage	Year of confirmation of outbreak through submission	NCBI	References
Algeria	2010	IV	2010	Yes	De Nardi et al. 2012
Angola	2012	IV	2012	No	Silva & Libeau 2014
Benin	1972	NA	NA	No	Bourdin 1973
	2016	II	2011	Yes	Adombi et al. 2017
Burkina Faso	NA	I	1988	Yes	Munir et al. 2012
	-	-	-	-	Kwiatek et al. 2011
	-	II	1999	No	Banyard et al. 2010
Cameroon	NA	IV	1997	Yes	Banyard et al. 2010
Central Africa	NA	IV	2004	Yes	Banyard et al. 2010
Chad	1971	II	1993	No	Provost, Maurice & Bourdin 1972
	-	-	-	-	Bidjeh et al. 1995
	-	-	-	-	Libeau et al. 2014
Comoros	2010	NA	NA	No	FAO 2013
Congo	NA	IV	2006	No	Muniraju et al. 2014
Democratic Republic of the Congo	2012	IV	2012	No	Salami et al. 2014
	-	-	-	-	Muniraju et al. 2014
Eritria	NA	IV	2002, 2003, 2005, 2011	Yes	Cosseddu et al. 2013
Ethiopia	1994	III	1994, 1996	Yes	Roeder et al. 1994
	-	-	-	-	Kwiatek et al. 2007
	-	-	-	-	Banyard et al. 2010
	-	IV	2010	Yes	Muniraju et al. 2014
Gabon	NA	IV	2011	Yes	Maganga et al. 2013
Ghana	NA	II	1976, 1978, 2010	Yes	Kwiatek et al. 2007
	-	-	-	-	Banyard et al. 2010
	-	-	-	-	Dundon et al. 2014
Guinea	NA	I	1988, 1991	Yes	Kwiatek et al. 2007
	-	-	-	-	Banyard et al. 2010
Guinea-Bissau	NA	I	1989	Yes	Kwiatek et al. 2007
Ivory Coast	1942	I	1989	Yes	Gargadennec & Lalanne 1942; Chard et al. 2008
Kenya	2006	III	2006	Yes	Wamwayi et al. 1995; Libeau et al. 2014
Libya	NA	NA	NA	No	Libeau et al. 2014; Almeshay et al. 2017
Mali	NA	II	1999	Yes	Kwiatek et al. 2007
Mauritania	NA	II	2012	Yes	El Arbi et al. 2014
Morocco	2008	IV	2008	Yes	Kwiatek et al. 2011; Parida et al. 2016
Niger	NA	II	2012	No	Farougou, Gagara & Mensah 2013; Libeau et al. 2014
Nigeria	1967	II	1975, 1976, 2010, 2012, 2013, 2014	Yes	Hamdy et al. 1976
	-	-	-	-	Diallo et al. 1994
	-	-	-	-	Shamaki 2002
	-	-	-	-	Chard et al. 2008
	-	-	-	-	Woma et al. 2016
	-	-	-	-	Mantip et al. 2016
	-	IV	2008, 2009, 2010,2012, 2013	Yes	Luka et al. 2012; Woma et al. 2016
	-	-	-	-	Mantip et al. 2016
Senegal	1955	I	1964, 1994	Yes	Mornet et al. 1956; Kwiatek et al. 2007
	-	II	2010, 2013	Yes	Banyard et al. 2010; Salami et al. 2014
Sierra Leone	2008	II	2009	Yes	Munir et al. 2012
Somalia	2006	III	NA	No	Nyamweya et al. 2009
Sudan	1971	III	1971, 1972, 2000	Yes	Ali & Taylor 1984
	-	-	-	-	Kwiatek et al. 2011
	-	-	-	-	Banyard et al. 2010
Tanzania	2008	III	2010, 2011, 2013,2015	Yes	Banyard et al. 2010
	-	-	-	-	Kgotlele et al. 2014
	-	-	-	-	Torsson et al. 2016
Togo	1972	NA	NA	No	Benazet 1973
Tunisia	NA	IV	2012, 2013	Yes	Sghaie et al. 2014
Uganda	1995	III	2007	No	Swai et al. 2009
	-	-	-	-	Banyard et al. 2010
	-	IV	2007, 2008	Yes	Luka et al. 2012; Muniraju et al. 2014
Western Sahara	NA	IV	2010	No	Libeau et al. 2014

*Source*: Parida, S., Muniraju, M., Mahapatra, M., Muthuchelvan, D., Buczkowski, H. & Banyard, A.C., 2015, ‘Peste des petits ruminants’, *Veterinary Microbiology* 181(1–2), 90–106. https://doi.org/10.1016/j.vetmic.2015.08.009.

Note: Lineages of isolates of Peste des petits ruminants virus were named by following the classification of lineages based on potential N gene sequence phylogenetic analysis.

NA, not available.

### Diagnosis and control of peste des petits ruminants in Africa

Peste des petits ruminants is recognised by farmers in Africa through the exhibition of profuse nasal discharge and diarrhoea by infected sheep and goats, mostly manifested during the onset of the rainy season and harmattan period. A presumptive diagnosis is usually based on clinical signs. Several diseases and conditions may however be confused with PPR in sheep and goats because of the similarity in clinical signs. These include rinderpest, bluetongue and contagious caprine pleuropneumonia. Diagnosis of the disease may also be complicated as a result of secondary bacterial infections specifically caused by *Mannheimia haemolytica*. Therefore, in addition to clinical observations, a differential diagnosis must be confirmed by laboratory diagnostic techniques. The laboratory tests currently available for diagnosis of the disease can be grouped into three categories: (1) those detecting virus or viral antigen (e.g. virus isolation, antigen capture ELISA and lateral flow devices), (2) those detecting genetic material from the virus (e.g. RT-PCR, real-time PCR and LAMP [loop-mediated isothermal amplification] PCR) and (3) those detecting antibodies against the virus (e.g. virus neutralisation test, competitive ELISA and indirect ELISAs). However, the efficiency of laboratory diagnosis can be greatly influenced by the integrity of the sample received, which is often affected by the conditions of the samples by collection and transportation.

There is no specific treatment against the disease, although a broad spectrum of antibiotics can be administered against secondary bacteria that could aggravate the disease condition because of the animal’s compromised immune status. Control of PPR in non-infected countries may be achieved using classical measures, such as the restriction of importation of sheep and goats from affected areas, isolation, quarantine, slaughter and proper disposal of carcasses and contact fomites and decontamination of affected premises in case of infection introduction. Control of PPR outbreaks can also rely on movement control (quarantine) combined with the use of focused ‘ring’ vaccination and prophylactic immunisation in high-risk populations. Homologous PPR vaccine attenuated after 63 passages in Vero cells (Adu et al. 1990; Diallo et al. 1989b) is presently used and can produce a solid immunity for 3 years (Libeau et al. 1995). The PPRV homologous vaccine was found to be safe under field conditions even for pregnant animals and it induced immunity in 98% of the vaccinated animals (Libeau et al. 1995). The PPR vaccine seed is available through the Pan African Veterinary Vaccine Centre (PANVAC) at Debre-Zeit, Ethiopia, for African countries. Recently, Mariner et al. (2017) developed a PPR thermostable vaccine using the rinderpest lyophilisation method to the attenuated Nigeria 75/1 PPR vaccine strain; the thermostability of the vaccine is sufficient enough to be used without a cold chain for up to 30 days, which will greatly facilitate the delivery of vaccination in the control, prevention and global eradication of PPR.

## Recommendations

A regional strategic vaccination campaign should be adopted across African countries. This can be achieved by the use of a PPR vaccine that is highly efficacious in protecting animals against all PPRV strains.

An epidemiological participatory technique, to assess livestock owners’ perceptions of vaccination success and serological surveys at a defined time period after vaccination to determine vaccinated animals’ immune response, should be adopted.

A periodic surveillance to understand the epidemiological situation to help define the status of PPR in Africa should be adopted. The purpose of this is to determine the early detection of the appearance of the disease or virus incursion; to demonstrate the absence of clinical disease or infection with PPRV; and to subsequently determine and monitor the prevalence, distribution and occurrence of the disease or infection across Africa.

Peste des petits ruminants causes an acute fatal disease in its natural host (sheep and goats), but a subclinical infection in cattle, buffaloes, camel and antelope. There is indeed a threat of the emergence of a new virulent morbillivirus of bovine and camelid, similar in manner to the way PPRV has emerged in sheep and goats. Host susceptibility, resistance and transmissibility to PPRV are not well understood. A systems biology approach through the mapping of the ‘infectious window’, that is, the period when an infected animal is able to infect other animals, which will be useful in unravelling the host factors associated with susceptibility, resistance and transmissibility to PPRV, should be carried out. This will contribute immensely in providing an instantaneous protection solution strategy and will equally contribute in avoiding unnecessary pre-emptive culling during epidemics.

## Conclusion

Because of the virulent nature of PPR in the majority of infected sheep and goats, most especially in naïve populations, even with the availability of an effective attenuated vaccine, PPR is still considered a disease of higher economic importance than other small ruminant diseases in PPR-endemic areas of Africa. Active surveillance for PPR is not carried out and the economic impact of the disease on the African livestock industry is thus difficult to precisely quantify. Nevertheless, not all farmers choose to vaccinate their animals and outbreaks of PPR amongst unvaccinated sheep and goats continue to occur on an annual basis. Clinical disease amongst unvaccinated sheep and goats in the field can be severe, and it is not uncommon for sheep and goat farmers in Africa to suffer significant losses amongst their flocks as a result of seasonal outbreaks of PPR. The campaign for the total eradication of PPR by FAO/OIE has stimulated the involvement of all the stakeholders in small ruminant production in the control, prevention and eventual eradication of PPR.
